# Cohort study of microelement status in “healthy” population of Russian megapolis

**DOI:** 10.1051/bmdcn/2019090315

**Published:** 2019-08-26

**Authors:** Andrew K. Martusevich, Konstantin A. Karuzin

**Affiliations:** 1 Privolzhsky Research Medical University, Nizhny Novgorod, Russia, Akapharm Ltd. Moscow

**Keywords:** Trace elements, Blood, Population, Megapolis

## Abstract

The aim of the study was at the cohort assessment of microelement status in large city residents classified as “apparently healthy people”. The population study included 2,025 randomly selected middle-aged (2045 years) persons without chronic diseases or acute infectious pathologies. The set of subjects was standardized by age and gender. A blood sample was taken once from each person to determine concentrations of microelements. The level of peripheral blood microelements were determined by atomic absorption spectrometry using «Shimadzu AA7000» device (Japan). The study of a large population of city residents demonstrated inhomogeneity in their microelement status. Deficit conditions were found for a number of values, including concentrations of potassium, sodium, nickel, stibium, chromium and cadmium. At the same time, there are large proportions of persons with low plasma concentrations of copper and even lower plasma concentrations of zinc and magnesium. On the contrary, 42% of the persons show high concentrations of lithium. Such disturbances of microelement homeostasis (pre-pathological condition) make it necessary to perform targeted correction for the purpose of preventing the development of pathological conditions associated with microelement deficiencies.

## 1. Introduction

Great public attention is currently focused on the problem of vitamin deficiency [1–4]. The latter is attributable, in particular, to nutritional habits, the environment and lifestyle in the world’s major cities [3, 5]. This situation can be corrected partially by prescribing various vitamins, promoting healthy lifestyle, including dietary principles [6–10]. The relevance of the problem is also confirmed by that the World Health Association has declared the current decade to be the Decade of Action on Nutrition (20112012) [11].

**Fig. 1:**
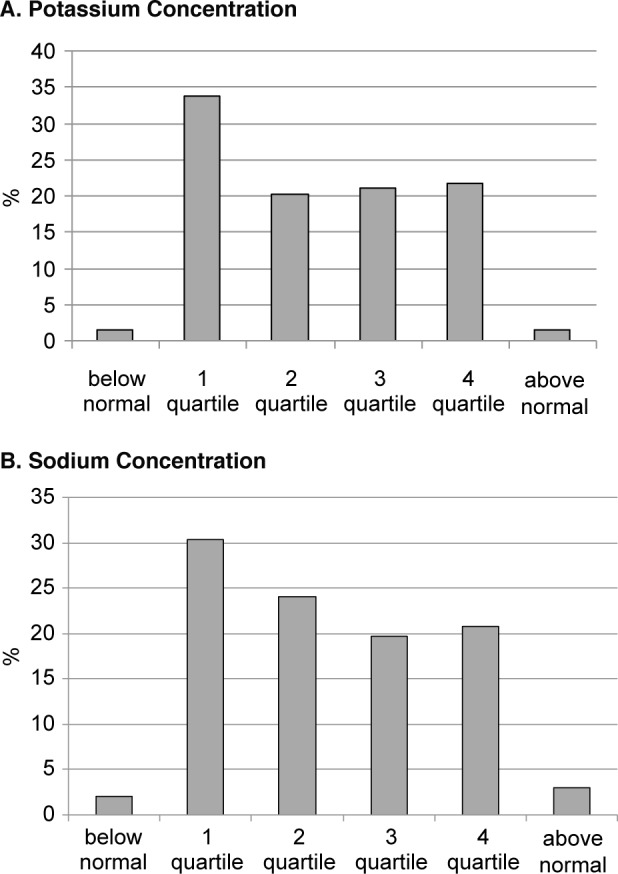
Blood concentrations of potassium and sodium in apparently healthy large city residents. (quartile structure)

**Fig. 2:**
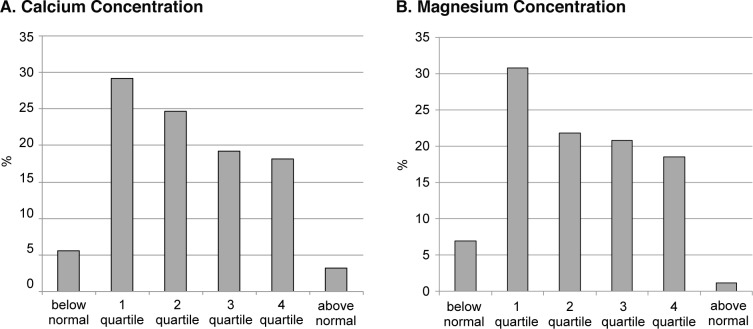
Blood concentrations of calcium and magnesium in apparently healthy large city residents. (quartile structure)

**Fig. 3:**
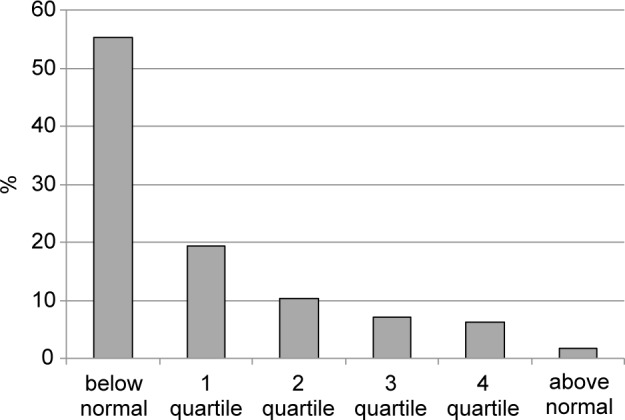
Plasma concentration of copper in large city residents. (quartile structure)

**Fig. 4:**
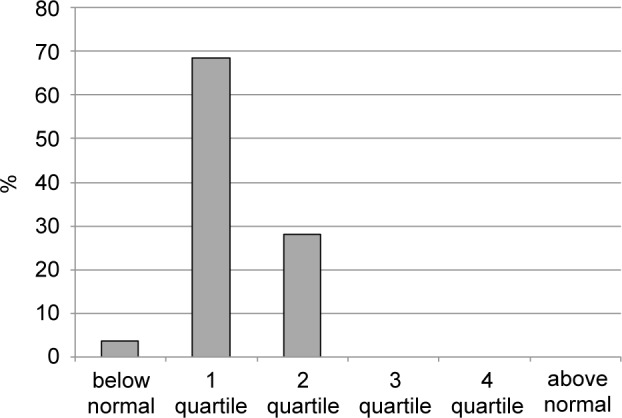
Plasma Concentration of Cadmium in Large City Residents. (quartile structure)

**Fig. 5:**
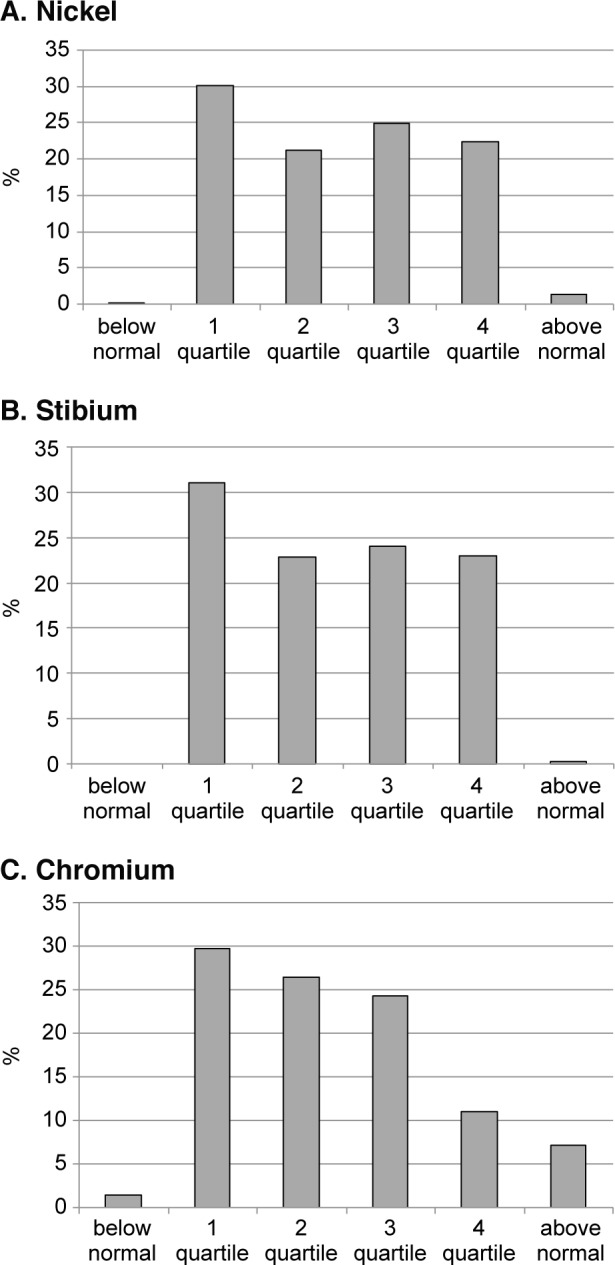
Blood concentrations of some microelements in apparently healthy large city residents. (quartile structure)

**Fig. 6:**
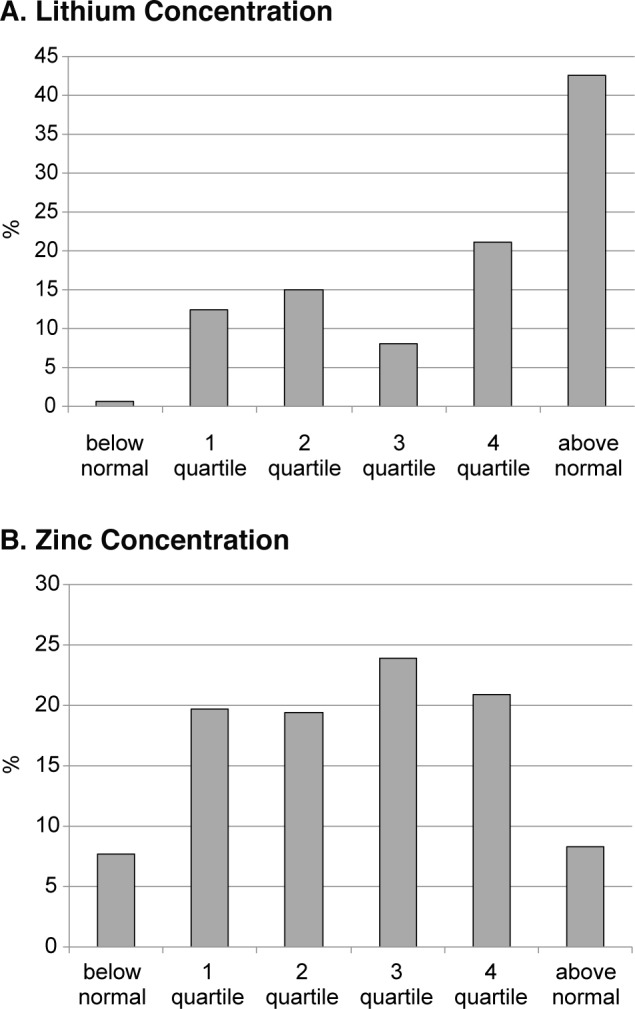
Plasma concentration of lithium in large city residents. (quartile structure)

**Table 1:** Level of some microelements in “healthy” population of megapolis. (n = 2,025)

Microelement	Plasma level, mcg/l
Potassium	169.76 ± 24.23
Sodium	3216.29 ± 261.87
Calcium	100.05 ± 6.59
Magnesium	22.09 ± 3.83
Copper	789.63 ± 223.68
Cadmium	0.92 ± 0.40
Nickel	0.55 ± 0.26
Stibium	0.24 ± 0.15
Chromium	13.15 ± 8.66
Lithium	32.40 ± 20.27
Zinc	960.54 ± 181.42

The other equally important but often neglected aspect of the general problem is the deficit of microelements, the role of which is significantly less known. At the same time, according to M. Houston (2008, 2010), both vitamin deficiency and microelement homeostasis disorders must be corrected concurrently [12, 13]. In order to solve the problem properly, full information on its status is required. However, there is only isolated data in world literature which relates primarily to evaluations of certain microelement concentrations in body fluids and tissues in particular diseases [1, 13]. In this case, they are generally pathogenetically associated with the related disease [1, 10, 14, 15]. Moreover, mineral homeostasis for a wide range of elements remains largely unstudied in residents of major cities who identify themselves as “apparently healthy people” [12, 13, 16–18]. On the other hand, people in this category are traditionally exposed only to superficial examination, mostly relating to the monitoring of vitamin and mineral homeostasis. From the perspective of preventive medicine, it is advisable for this segment of the population to detect and timely correct prenosological disorders for the purpose of their timely individual correction and, thus, preventing the development and progression of microelementosis [12, 14, 19, 20].

In this regard, the work is aimed at the cohort assessment of microelement status in large city residents classified as “apparently healthy people”.

## 2. Methods

The population study was conducted with 2,025 randomly selected persons. We included in this study middle-aged (20-45 years) peoples. Exclusion criterias was an absence of chronic diseases or acute infectious pathologies. The set of subjects was standardized by age and gender. All the patients signed the informed consent for inclusion in this study.

A blood sample was taken once from each person to determine concentrations of microelements. All subjects were tested during the morning hours. The level of peripheral blood microelements were determined by atomic absorption spectrometry using «Shimadzu AA7000» device (Japan).

The study was approved by a local Ethics Committee of the Privolzhsky Federal Medical Research Center (N°31; 14/12/2015).

Statistical analysis was performed by means of variation statistics. Mean and standard deviations were calculated for each parameter. At the next stage, as per the valid standard values of the certified laboratory, the region of values was divided into 6 ranges: below normal, quartiles 1 to 4 (Q1 = 0-25% of reference interval; Q2 = 26-50%; Q3 = 51-75%; Q4 = 76-100%), above normal. The data was expressed as a percentage for each of the selected ranges.

## 3. Results and discussion

It is interesting to note that the average plasma concentrations of all studied microelements, except for lithium, did not deviate markedly from the physiological standard (Table 1). At the same time, the quartile analysis of the microelement status in the large city population established that the distribution patterns differ significantly from the a priori expected Gaussian distribution by a large number of parameters.

It is important to emphasize that this tendency fully applies not only to microelements but also to the elements classified as principal biogenic ones. So, the distribution pattern of the tested persons by potassium concentration showed that more than a third of the patients (33.7%) were at the lower limit of normal (quartile 1), which indicates latent hypokaliemia (Fig. 1A).

A similar situation is observed for the blood concentration of sodium, although its shift is smoother as compared to the distribution pattern for blood potassium (Fig. 1B). Nevertheless, hyponatremia and the level close to it (quartile 1) are recorded in 32.5% of the tested apparently healthy adult citizens.

As for other rare earth metals (calcium and magnesium), lower values were also registered in most cases (Fig. 2), and greater percentages of persons were in a deficiency state (5.6% and 7%, respectively). Furthermore, the values were lower in about a third of the tested persons (29.2% for calcium and 30.8% for magnesium). Taking into account wide-ranging biological functions of these elements, their deficit can be considered as a pre-pathological condition which requires targeted individual correction.

Our studies established that there are also deficiencies of a number of microelements. Thus, over a half of the tested persons (55.3%) show decreased concentration of copper, and 14% of subjects fall into quartile 1 for this value, showing the pre-deficit condition (Fig. 3). Being a component of a wide range of enzymes, this element belongs to the category of biogenic elements, which makes it necessary to correct its concentration as well.

An even more pronounced disturbance of microelement homeostasis was registered for cadmium concentration (Fig. 4). For this element, all tested large city residents showed a deficit (3.6%) or quartile 1-2 (68.5% and 27.9%, respectively), not exceeding the median reference interval.

A less pronounced downshift disturbance of the distribution pattern was also observed for other microelements, such as nickel, stibium and chromium (Fig. 5). The most pronounced shift among them was registered for chromium concentration, with chromium deficiency and decreased value (quartile 1) found in 31.2% of the persons tested (Fig. 5C). In opposite, we fixed that a significant part of tested population (about 50%) has high concentration of nickel and stibium in blood plasma (3 and 4 quartile or above normal value).

This may indirectly indicate man-made pollution in a large megapolis. This trend is more pronounced in the plasma level of nickel, in which a larger proportion of healthy people had a value above normal.

It is interesting that there is a reverse tendency for some microelements showing increased values. In particular, over 42% of the large city population is characterized by high lithium concentrations, and another 21.2% of the tested persons show a tendency towards an increase in the concentration of this element (Fig. 6A). A similar but much more smoothed pattern was registered for plasma zinc concentration, although for this parameter, a significant proportion of the population (7.7%) has hypozincemia, which can be considered as a pre-pathological condition.

## 4. Conclusion

The study of a large population of city residents demonstrated inhomogeneity in their microelement status. Deficit conditions were found for a number of values, including concentrations of potassium, sodium, nickel, stibium, chromium and cadmium. At the same time, there are large proportions of persons with low plasma concentrations of copper and even lower plasma concentrations of zinc and magnesium. On the contrary, 42% of the persons show high concentrations of lithium. Such disturbances of microelement homeostasis (pre-pathological condition) make it necessary to perform targeted correction for the purpose of preventing the development of pathological conditions associated with microelement deficiencies. In this regard, our further research will be focused on evaluating the effectiveness of personalized correction of micronutrient deficiencies in various metabolic parameters.

## Conflicts of interest statement

The authors wish to disclose no conflicts of interest.
